# Supplementary material for: OpenMM 7: Rapid development of high performance algorithms for molecular dynamics
Source: PLoS Comput Biol. 2017 Jul 26;13(7):e1005659. doi: 10.1371/journal.pcbi.1005659 (PMC5549999; doi:10.1371/journal.pcbi.1005659)
Supplement: S1 Source Code — (ZIP) [file pcbi.1005659.s002.zip › OpenMM-7.0.1-Source/docs/api-c++/generated/OpenMM.Vec3.html]

Vec3 — OpenMM @OPENMM\_MAJOR\_VERSION@.@OPENMM\_MINOR\_VERSION@ documentation


# Vec3¶

*class* `OpenMM::``Vec3`¶
:   This class represents a three component vector. It is used for storing positions, velocities, and forces.

    Methods

    |  |  |
    | --- | --- |
    | `Vec3` | Create a `Vec3` whose elements are all 0. |
    | `Vec3` | Create a `Vec3` with specified x, y, and z components. |
    | `operator[]` |  |
    | `operator[]` |  |
    | `operator==` |  |
    | `operator!=` |  |
    | `operator+` |  |
    | `operator+` |  |
    | `operator+=` |  |
    | `operator-` |  |
    | `operator-` |  |
    | `operator-=` |  |
    | `operator*` |  |
    | `operator*=` |  |
    | `operator/` |  |
    | `operator/=` |  |
    | `dot` |  |
    | `cross` |  |

    `Vec3`()¶
    :   Create a `Vec3` whose elements are all 0.

    `Vec3`(double *x*, double *y*, double *z*)¶
    :   Create a `Vec3` with specified x, y, and z components.

    double `operator[]`(int *index*) *const*¶

    double &`operator[]`(int *index*)¶

    bool `operator==`(*const* Vec3 &*rhs*) *const*¶

    bool `operator!=`(*const* Vec3 &*rhs*) *const*¶

    Vec3 `operator+`() *const*¶

    Vec3 `operator+`(*const* Vec3 &*rhs*) *const*¶

    Vec3 &`operator+=`(*const* Vec3 &*rhs*)¶

    Vec3 `operator-`() *const*¶

    Vec3 `operator-`(*const* Vec3 &*rhs*) *const*¶

    Vec3 &`operator-=`(*const* Vec3 &*rhs*)¶

    Vec3 `operator*`(double *rhs*) *const*¶

    Vec3 &`operator*=`(double *rhs*)¶

    Vec3 `operator/`(double *rhs*) *const*¶

    Vec3 &`operator/=`(double *rhs*)¶

    double `dot`(*const* Vec3 &*rhs*) *const*¶

    Vec3 `cross`(*const* Vec3 &*rhs*) *const*¶

High performance molecular simulation on GPUs


### Navigation

- Core Objects
- Forces
- Integrators
- Extras

©2015, Stanford University and the Authors.
|
Powered by Sphinx 1.3.1
& Alabaster 0.7.7
|
Page source
